# Use of Long Short-Term Memory for Remaining Useful Life and Degradation Assessment Prediction of Dental Air Turbine Handpiece in Milling Process

**DOI:** 10.3390/s21154978

**Published:** 2021-07-22

**Authors:** Yi-Cheng Huang, Yu-Hsien Chen

**Affiliations:** Department of Mechatronics Engineering, National Changhua University of Education, Changhua 50074, Taiwan; hjnm4512159@gmail.com

**Keywords:** dental air turbine handpiece, long short-term memory, logistic regression, remaining useful life

## Abstract

The complexity of the internal components of dental air turbine handpieces has been increasing over time. To make operations reliable and ensure patients’ safety, this study established long short-term memory (LSTM) prediction models with the functions of learning, storing, and transmitting memory for monitoring the health and degradation of dental air turbine handpieces. A handpiece was used to cut a glass porcelain block back and forth. An accelerometer was used to obtain vibration signals during the free running of the handpiece to identify the characteristic frequency of these vibrations in the frequency domain. This information was used to establish a health index (HI) for developing prediction models. The many-to-one and many-to-many LSTM frameworks were used for machine learning to establish prediction models for the HI and degradation trajectory. The results indicate that, in terms of HI predicted for the testing dataset, the mean square error of the many-to-one LSTM framework was lower than that that of a logistic regression model, which did not have a memory framework. Nevertheless, high accuracies were achieved with both of the two aforementioned approaches. In general, the degradation trajectory prediction model could accurately predict the degradation trend of the dental handpiece; thus, this model can be a useful tool for predicting the degradation trajectory of real dental handpieces in the future.

## 1. Introduction

Dental air turbine handpieces are the most common medical equipment used by dentists for treating teeth. The main mechanical mechanism of the aforementioned equipment involves the introduction of high-pressure air into the head to rotate microturbine blades for driving bearings to rotate a tooth drill. Before or during treatment, a dentist cannot identify the state of health (SOH) of and the presence of any damage in the internal components of a dental handpiece. Because a dental handpiece has a high rotation speed, its internal bearing gradually degrades and becomes damaged after it has been used for a long time. Consequently, ceramic balls cannot operate stably along the designated track, which results in increased internal friction in the shell of the machine head and in increased temperature. Heat is transferred to the bur through the rotor, which increases the temperature of the bur. When the temperature of the bur at the milling contact surface is higher than 42.5 °C [1], it may cause irreversible damage to teeth. When the temperature reaches 52 °C, it may cause pulp necrosis. The vibrations of the bur make the patient uncomfortable and cause the tooth to have an uneven surface.

The authors of [2] used an accelerometer and a laser Doppler vibrometer to measure vibration signals during the operation of a dental handpiece. By comparing the spectrograms of the handpiece without loading (free running) and with loading (milling teeth), the aforementioned authors discovered that the vibration frequency decreased during milling. The two measurement results did not exhibit significant differences. The authors of [3,4] used different operating conditions of dental air turbine handpieces to generate an experimental group and a control group. Dental handpieces were used according to pre-established testing procedures until their bearing failed, through which they analyzed factors affecting the service life of the bearing. The authors of [4] mentioned that the failure of the ball bearing was typically caused by faults in the cage (the nonmetal part of the bearing). When the bearing fails, the cage experiences substantial damage due to the running of the instrument under load and the occurrence of corrosion in high-pressure sterilization. Although it cannot be determined whether the wear and fracture of the cage directly leads to bearing faults, damage to the cage unavoidably hinders the stable operation of the ball in the raceways, which is sufficient to cause damage to the ball and raceways under relatively few rotations. Therefore, frictional heat is generated, which causes the formation of a heat-affected zone. When a dentist uses the handpiece, this heat is unavoidably conducted to the tooth drill.

Therefore, establishing a prediction model for the health index (HI) of dental handpieces is critical. Such a model can be used to monitor the SOH of a dental handpiece and predict its remaining useful life (RUL) in real time, which can allow dentists to identify the timing of potential equipment faults on the basis of comprehensive factors such as internal bearing damage, cage fracture, or loss of original dynamic balance by the rotor. Moreover, a dentist can use the aforementioned information to determine the time for replacing parts of the dental handpiece, such as the bearing, in the next usage cycle so that the handpiece does not suddenly malfunction during treatment and injure the patient.

In [5], the diagnostic results of a free-running of an air turbine dental handpiece (ATDH) with three rotor statuses by applying fast Fourier transform (FFT), Hilbert–Huang transform (HHT), and multiscale entropy (MSE) processes was studied. Our previously proposed method was tested under conditions of additional axial preload on the rotor and ceramic bearings with a damaged outer race supporting the rotor. Using a laser-Doppler vibrometer, condenser microphone, and portable MEMS system microphone were used to acquire the signals when the ATDH rotor features were changed. The results show that changes in preload or malfunctioning ball bearings can be discriminated and abstracted using FFT and HHT to analyze the vibration frequencies with three different sensing devices. However, no study has investigated the RUL of dental handpieces. Therefore, the current study used a long short-term memory (LSTM) network to establish an HI prediction model. Because an LSTM network can transfer memory and learn time-series data, this network is suitable for fault prediction and prognostic and health management, such as monitoring the system status and performing predictive maintenance.

Several studies have used different methods to construct an RUL system. The authors of [6,7] predicted the RUL of the bearing by using the moving-average cross-correlation based on power spectral density, a deep autoencoder, and a deep neural network. The authors of [8] used different RUL models for friction systems under different operation conditions. By using the signal trend and predefined threshold values, the aforementioned authors identified which model to use and estimated the RUL, thereby increasing the accuracy of the estimation. With advances in machine learning, studies have applied logistic regression (LR) to assess the degradation of machines. In [9], the wavelet packet decomposition technique was used to obtain features from data, such as current and vibration data, select critical features, and enter these features into the LR model for assessing machine performance and identifying possible failure models. With increases in the calculation speed of computers, deep learning has been applied practically in fields such as medicine and engineering. Numerous studies have adopted deep learning in prognostics and health management, including fault diagnosis and RUL prediction. In [10,11], the SOH and RUL of Li batteries were assessed. In [10], features were obtained from the incremental capacity curve to analyze the correlation between the features and the battery capacity. This analysis was conducted to select features with high correlations as the input for artificial neural network (ANN) assessment models. Moreover, the percentages of the current capacity and initial capacity were used as standards to train assessment models to determine the SOH of lithium-ion batteries. Finally, the testing results of the validation dataset indicated that the ANN assessment models had high accuracy. Convolutional neural networks (CNNs) and recurrent neural networks (RNNs), which are two types of deep learning algorithms, have been widely used in various fields. CNNs are commonly used for handling classification problems such as image recognition and bearing fault diagnosis [12]. By contrast, RNNs are commonly used for handling time series problems. Because RNNs use the output from the hidden layer for the previous moment as the input of the hidden layer for the next moment, the output of each moment is associated with the input of the previous moment. Thus, RNNs can learn from and memorize time series. However, RNNs face the exploding gradient and vanishing gradient problems [13], rendering them difficult to train. LSTM networks can solve the vanishing gradient problem because, unlike in RNNs, in which the memory of each instance is replaced by a new memory, the previous memory is added to the new memory in LSTM networks. In [14], an LSTM model was used to predict oil prices. In [15,16], LSTM models were used in time-series-related research, such as RUL assessment. In [16], RUL indicators of turbofan engines were generated using LSTM models. Data obtained from sensors that exhibited relatively stable changes with the degradation of the engine were used in the aforementioned study. These data were subjected to processing procedures, such as filtration and normalization. Compared with linear decreasing, the physical signals obtained from the sensors could more accurately reflect the actual degradation of engines. Because the real SOH and RUL of a system are generally unknown, a piece-wise linear decreasing function was used in [17] to determine the RUL. For a healthy system, degradation is not obvious; therefore, the authors of [17] assumed that the RUL is initially constant and then decreases linearly. This study used an accelerometer to obtain the vibration signals of dental handpieces for analyzing the signal trends and features generated as the rotor of the dental handpieces gradually degraded with time. The identified trends and features were used as a critical degradation index to develop an HI model for assessing the SOH and RUL of dental handpieces.

## 2. Method

### 2.1. Many to One and Many to Many LSTM Structures for RUL and Degradation Assessment

**Figure 1:**
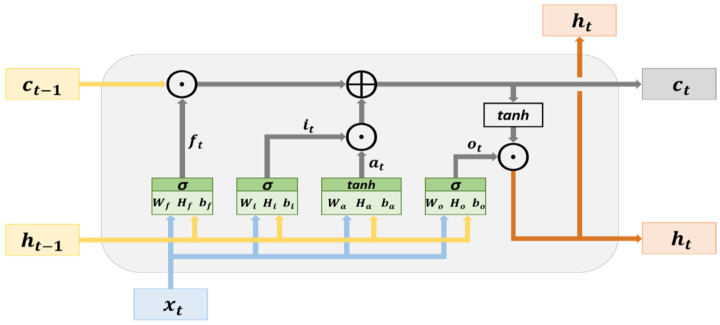
Architecture of LSTM memory cells.

An LSTM possesses a memory structure that contains memory cells. It adds and memorizes information as the time series progresses, thereby solving the vanishing gradient problem. Figure 1 illustrates the basic structure of an LSTM network. The cell state can be used to store and transmit memory; thus, the information in this state can only be written or deleted. Without external influence, the aforementioned information remains unchanged. The parameter $x_{t}$ represents the input data at time t, and $h_{t-1}$ is the hidden state at time t−1. The cell state at time t−1 is denoted as $C_{t-1}$, which is modified to the present cell state $\;C_{t}$ in the hidden layer at time t.

The hidden layer of an LSTM network contains an input node ($a_{t}$) and three controlled gates ($f_{t},\;\;i_{t},\;\;\mathrm{and}\;o_{t}$). The variables $a_{t}$, $f_{t},\;\;i_{t},\;\;\mathrm{and}\;o_{t}$ are calculated using (1)–(4), respectively. The input node $a_{t}$ is used for updating the cell state, whereas the controlled gates are used to determine whether to allow information to pass through them. The controlled gates are the forget gate, input gate, and output gate. The forget gate ($f_{t}$) determines which cell states’ ($c_{t-1}$) information may pass through it. The input gate ($i_{t}$) determines which input nodes’ information ($a_{t}$) may pass through it. The vectors (information) passing through the input gate are used for updating the cell state and are subjected to element-wise addition with the vectors (information) passing through the forget gate to generate the cell state at time t ($c_{t}$). The calculation in the aforementioned process is expressed in (5). The output gate determines which cell states’ ($c_{t}$) information may pass through it. The vectors (information) passing through the output gate are in the hidden state at time t ($h_{t}$), and they are the output vectors of the current hidden layer. The calculation method for $h_{t}$ is presented in (6). In addition, the cell state and hidden state obtained at time t, namely ($c_{t}$) and ($h_{t}$), respectively, are transmitted to the hidden layer at time (t+1). This process that progresses with the time series is used for the transmission and learning of memory.

(1) $$a_{t}=tanh\left(W_{a}x_{t}+H_{a}h_{t-1}+b_{a}\right)$$

(2) $$f_{t}=\sigma \left(W_{f}x_{t}+H_{f}h_{t-1}+b_{f}\right)$$

(3) $$i_{t}=\sigma \left(W_{i}x_{t}+H_{i}h_{t-1}+b_{i}\right)$$

(4) $$o_{t}=\sigma \left(W_{o}x_{t}+H_{o}h_{t-1}+b_{o}\right)$$

(5) $$c_{t}=\left(f_{t}⊙c_{t-1}\right)⊕\left(i_{t}⊙a_{t}\right)$$

(6) $$h_{t}=o_{t}⊙tanh\left(c_{t}\right)$$

where W and H represent the weight, b denotes the bias, and ⊕ is the symbol for element-wise addition, ⊙ is the symbol for element-wise multiplication, tanh denotes the hyperbolic tangent, and σ represents the sigmoid function. The parameters tanh and σ represent activation functions.

**Figure 2:**
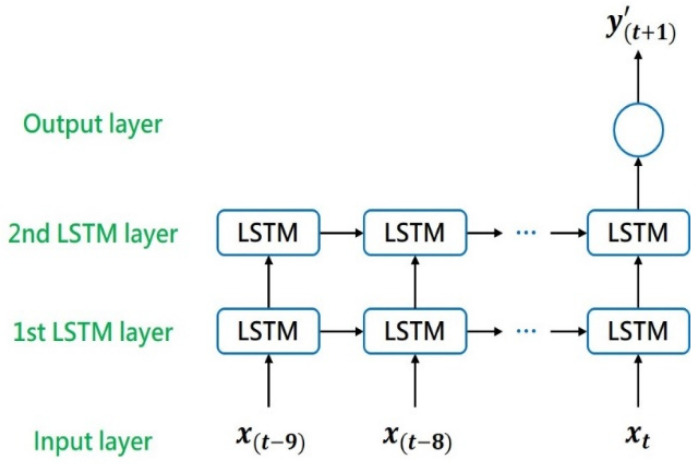
LSTM model with the many-to-one structure.

The current study adopted the many-to-one and many-to-many LSTM structures. These structures were used to develop different prediction models. When the current state was known, a model based on the many-to-one LSTM structure was used to predict the SOH of the next unknown cycle for the early assessment of the SOH of a dental handpiece. Consider the example of ten time steps. SOH data of the current known cycle and the nine cycles before it, namelexample of ten time steps. SOH data of the current known cycle and the nine cycles before it, namely [$x_{\left(t-9\right)},x_{\left(t-8\right)},x_{\left(t-7\right)}$, …, $x_{\left(t\right)}$], are entered into the LSTM model, which then outputs the SOH of the next unknown cycle, namely [$y_{\left(t+1\right)}^{’}$]. Figure 2 illustrates an LSTM model with the many-to-one structure.

**Figure 3:**
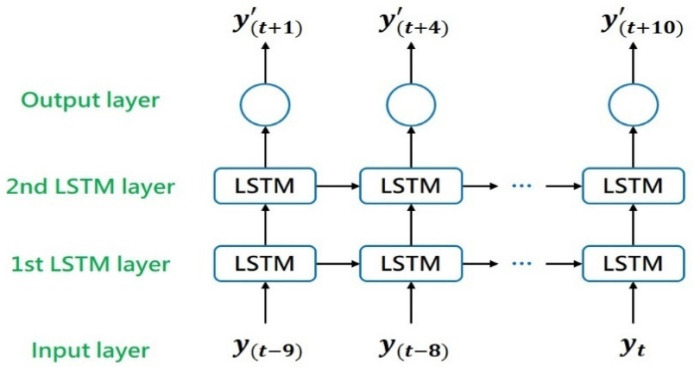
LSTM model with the many-to-many structure.

The many-to-many structure is used to predict the degradation trajectory of dental handpieces. Consider the example of ten time steps. The SOHs of the current known cycle and the nine cycles before it, namely [$y_{\left(t-9\right)},y_{\left(t-8\right)},y_{\left(t-7\right)}$, …, $y_{\left(t\right)}$], are input into the LSTM model, which then output the SOHs of the next ten unknown cycles, namely [$y_{\left(t+1\right)}^{’},y_{\left(t+2\right)}^{’},y_{\left(t+3\right)}^{’}$, …, $y_{\left(t+10\right)}^{’}$]. Subsequently, the output results are used as the model input. The aforementioned model then outputs the SOHs of the next ten unknown cycles, namely [$y_{\left(t+11\right)}^{’},y_{\left(t+12\right)}^{’},y_{\left(t+13\right)}^{’}$, …, $y_{\left(t+20\right)}^{’}$]. This method is repeated for iteration to predict the degradation trajectory of a dental handpiece. Figure 3 shows an LSTM model with the many-to-many structure. In the many-to-one structure, in each time step, the model can accept multidimensional input data. By contrast, in the many-to-many structure, in each time step, input data must be unidimensional to enable the model to continue iterating.

### 2.2. Logistic Regression Prediction Model for Many to One Structure

In Equation (7), the sigmoid function is used for predicting the output of LR with the inputs features $x_{1}–x_{n}$ in Equation (8) using model parameters $\theta_{0}–\theta_{n}$. A conventional run-to-failure LR sets y as a constant to 1 for the initial healthy status, to 0 for failure, and to 0–1 for the rest of the work asset’s lifetime. To build the RUL model accurately with a LR, the difference between the predicted $h_{\theta }\left(x\right)$ and y is minimized. Therefore, the model parameters for each $\theta_{0}–\theta_{n}$ are trained based on Equation (7) to predict the actual CV, *y*, of the machine’s health. The model parameters for $\theta_{0}$–$\theta_{n}$ can be derived using the Bernoulli distribution, maximum likelihood estimation (MLE), a loss function, and the method of gradient descent.

(7) $$h_{\theta }\left(x\right)=\frac{1}{1+e^{-\theta^{T}x}}$$

(8) $$\theta^{T}x=\theta_{0}+\theta_{1}x_{1}+\theta_{2}x_{2}+\theta_{3}x_{3}+\ldots +\theta_{n}x_{n}$$

The probability by Bernoulli distribution is given by Equation (9).

(9) $$p\left(y|x\right)=h_{\theta }{\left(x\right)}^{y}{\left(1-h_{\theta }\left(x\right)\right)}^{1-y}=\{\begin{array}{c} h_{\theta }\left(x\right)\;\;\;\;\;\;\;\;\;\;\;\;\;\;\;if\;y=1\\ 1-h_{\theta }\left(x\right)\;\;\;\;\;\;\;if\;y=0 \end{array}$$

The MLE function is applied by multiplying each probability in Equation (9) after the most likely real outputs *y* are obtained from the inputs $X_{1}–X_{m}$ and the model parameters $\theta_{0}–\theta_{m}$. Thus, Equation (10) provides a likelihood estimation function based on m known samples and the loss function in Equation (11). Iterations of the gradient descent method with the learning rate (α) to find the value of each model parameter $\theta_{j}$ are performed according to Equations (12) and (13).

(10) $$L\left(\theta \right)=\overset{m}{\underset{i=1}{\prod }}p\left(y^{\left(i\right)}|x^{\left(i\right)};\theta \right)=\overset{m}{\underset{i=1}{\prod }}h_{\theta }{\left(x^{\left(i\right)}\right)}^{y^{\left(i\right)}}{\left(1-h_{\theta }\left(x^{\left(i\right)}\right)\right)}^{1-y^{\left(i\right)}}$$

(11) $$J\left(\theta \right)=-\log L\left(\theta \right)=\overset{m}{\underset{i=1}{\sum }}\left(\log\left(1+exp^{\theta^{T}x^{\left(i\right)}}\right)-y^{\left(i\right)}\theta^{T}x^{\left(i\right)}\right)$$

(12) $$\frac{\partial J\left(\theta \right)}{\partial \theta_{j}}=\frac{\partial \sum_{i=1}^{m}\left(\log\left(1+exp^{\theta^{T}x^{\left(i\right)}}\right)-y^{\left(i\right)}\theta^{T}x^{\left(i\right)}\right)}{\partial \theta_{j}}=\overset{m}{\underset{i=1}{\sum }}\left(h_{\theta }\left(x^{\left(i\right)}\right)-y^{\left(i\right)}\right)x_{j}^{\left(i\right)}$$

(13) $$\theta_{j}=\theta_{j}-\alpha \overset{m}{\underset{i=1}{\sum }}\left(h_{\theta }\left(x^{\left(i\right)}\right)-y^{\left(i\right)}\right)x_{j}^{\left(i\right)}$$

**Figure 4:**
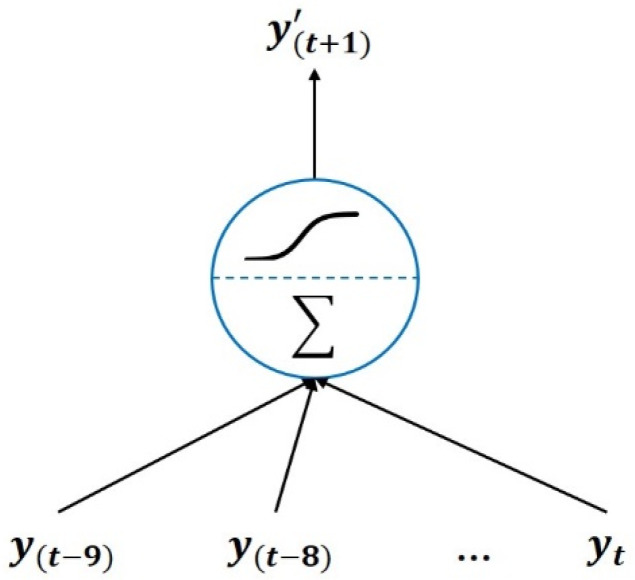
Illustration for LR model.

The LR structure is used to predict the SOH of the next unknown cycle on the basis of the current known state; thus, the LR structure can achieve the early assessment of the SOH of a dental handpiece. Because the LR structure does not possess the cell state of the LSTM network for storing and transmitting memories, the LR structure directly transmits the known state into the model. Consider the example of ten time steps. SOH data of the current state and the nine cycles before it, namely [$y_{\left(t-9\right)},y_{\left(t-8\right)},y_{\left(t-7\right)}$, …, $y_{\left(t\right)}$], are input simultaneously into the LR model, which then outputs the SOH of the next unknown cycle, namely [$y_{\left(t+1\right)}^{’}$]. The structure of the LR model is displayed in Figure 4. The current study compared the LSTM model, which possesses the memory storage and transmission functions, with the LR model, which does not possess these functions, and analyzed their prediction results.

### 2.3. Kalman Filter

A Kalman filter (KF) combines the least square method and state-space representation in the recursive solution of linear filtering for a dynamic system. The KF is one of the optimal recursive algorithms in data processing, particularly in the field of navigation, communication, and satellite and flight control, where high tolerance to noise is crucial. Contrary to the ordinary concept of low-pass, high-pass, and band-pass filters, the KF is an estimation and prediction approach based on a probability density function. The KF, with the optimal recursive data processing algorithm, can estimate the present state by estimating the previous step and observing the current state. Therefore, without adopting previous observations and estimation data, less memory space is used, and a shorter system response time can be obtained. Furthermore, the KF outperforms its low- and high-pass counterparts in handling probabilistic noises.

During execution, operations of the KF can be divided into two parts, namely prediction and correction. In the recursive process, the following notations are employed [18]:

${\tilde{x}}_{k}$: posteriori estimate vector at time step k

$A_{k}$: state transition matrix at time step k, $A\in R^{n\times n}$

$B_{k}$: input control matrix at time step k, $B\in R^{n\times 1}$

$z_{k}$: measurement vector at time step k

$H_{k}$: measurement matrix at time step k

$v_{k}$: measurement noise at time step k

$Q_{k}$: process noise covariance matrix

$R_{k}$: measurement noise covariance matrix

$Q_{k}$ and $R_{k}$ are the influences of the external disturbance noise and measurement noise, respectively, on the system. The recursive process of the KF can be explained as follows:

I. Prediction

State prediction:

(14) $${\tilde{x}}_{k}^{-}=A_{k}{\tilde{x}}_{k-1}+B_{k}u_{k-1}$$

Error covariance matrix prediction:

(15) $$P_{k}^{-}=A_{k}P_{k-1}A_{k}^{T}+Q_{k}$$

II. Correction

State correction:

(16) $$K_{k}=P_{k}^{-}H_{k}^{T}{\left(H_{k}P_{k}^{-}H_{k}^{T}+R_{k}\right)}^{-1}$$

(17) $${\tilde{X}}_{k}={\tilde{x}}_{k}^{-}+K_{k}(z_{k}-H_{k}{\tilde{x}}_{k}^{-})$$

(18) $$P_{k}=(1-K_{k}H_{k})P_{k}^{-}$$

In the aforementioned procedure, estimation and prediction using the previous time step can be employed for estimating and predicting the subsequent time step; that is, the previous state is used to determine the subsequent state. In this study, the state transition matrix is performed as a single variable at a time for filtering.

## 3. Experiments

### 3.1. Experimental Setup and Milling

**Figure 5:**
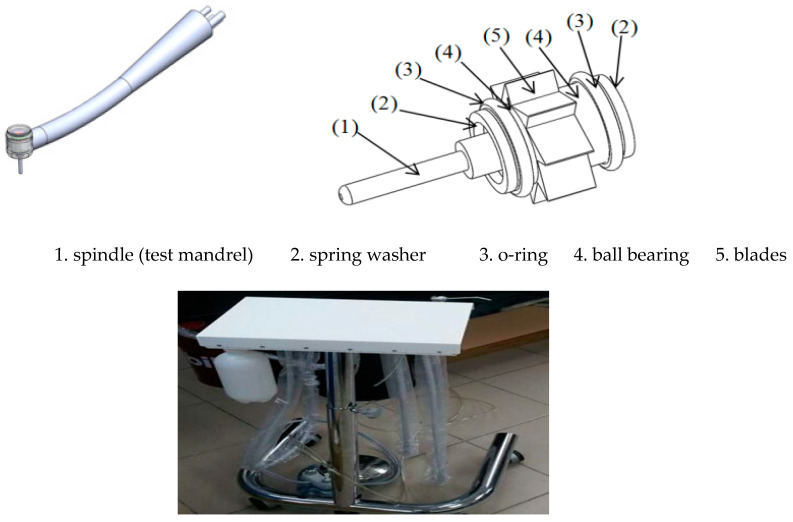
Schematic illustration and picture of in-house ATDH measurement platform.

**Figure 6:**
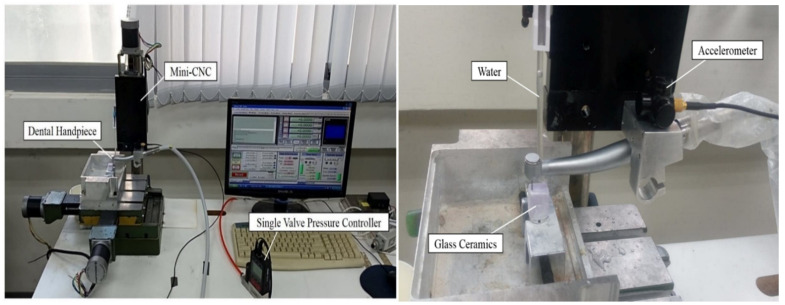
Triaxial CNC dental handpiece used in the cutting experiment.

**Figure 7:**
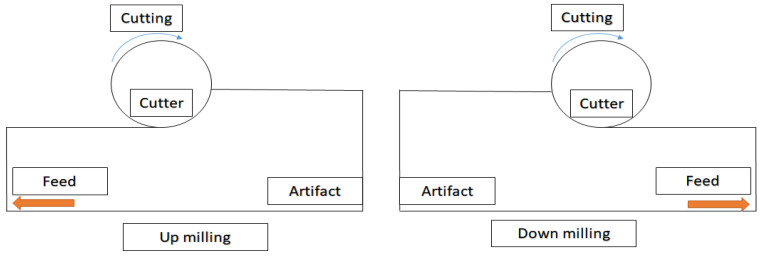
Schematics of up-milling (**left**) and down-milling (**right**).

**Figure 8:**
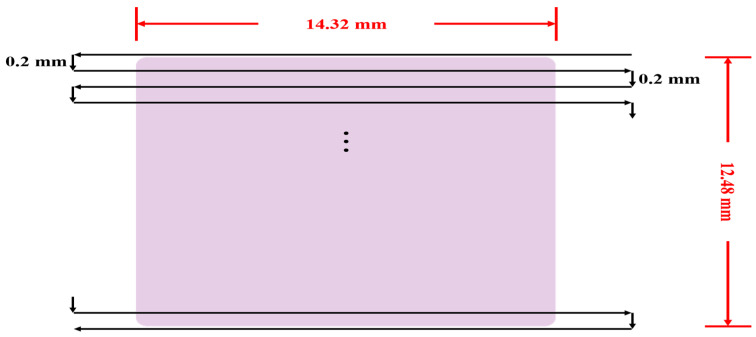
Schematic of the cutting path for one cycle.

The experimental instruments consisted of a dental device platform and a dental air turbine handpiece (Figure 5). The dental air turbine handpiece (Tiger101-3T4, 300 × 10^3^–360 × 10^3^ rpm, Thunder, Tiger group, Taichung, Taiwan) contained a machine head, hand grip, and handpiece connector. The adopted carbide bur (FG 558) had a diameter of 1.0 mm and a total length of 19 mm. This study used an Alicat PC-Series proportional–integral–derivative (PID) single-valve pressure controller to control the drive air pressure (Figure 6). An RS-232 signal line was connected to the Flow Vision SC software program on a personal computer. On the computer, various parameters related to the pressure control valve were adjusted to determine the location of this valve and the input voltage. The input voltage was adjusted to control the set point. A triaxial desktop computer numerical control (CNC) machine (mini-CNC P RX 1510, Original Mind Co., Nagano, Japan) was used to simulate the motions of humans operating the dental handpiece. This machine was used to move the dental handpiece and a glass ceramic block to conduct a cutting experiment. The material used for cutting was glass ceramic tempered with IPS e.max CAD. A triaxial accelerometer (sensitivity: 1000 mV/g) was glued to a jig. The NI  cDAQ-9174 and NI 9230 instruments were used to measure and capture the vibration signals of the handpiece during free running. The collected data were transferred to and stored in LabVIEW. The sampling frequency was set at 12.8 kHz. The overall experimental setup is illustrated in Figure 6. The cutting conditions were as follows: feed rate = 100 mm/min, air pressure = 50 psi, and cutting depth = 0.1 mm. The cutting process involved linear back-and-forth milling (up- and down-milling; Figure 7). The cutting width was 0.2 mm until the glass ceramic was cut to a depth of 0.1 mm, which was the cutting path of a cycle, as illustrated in Figure 8. After completing the cutting path of a cycle, the vibration signals of the dental handpiece during free running were obtained during the cycle. In this manner, free-running vibration signals were obtained for different cycles. In each cycle, a volume of approximately 17.87 ${\mathrm{mm}}^{3}$ was removed. Each cycle lasted approximately 20 min. Excluding the factor of mill wear, a new tooth drill (based on several experimental experiences) could generally be used for 20 cycles. Then, according to the visual inspection results, the tooth drill was replaced.

Two new dental handpieces were used in the experiment. After dental handpiece No. 1 completed 250 cycles (5000 min) of the cutting experiment, it did not reflect clear degradation characteristics in the time- or frequency-domain analyses. Considering the reliability factors of handpiece production, the cutting experiment was continued. To quicken the degradation of the aforementioned dental handpiece, after approximately 300 cycles, the conditions of the cutting experiment were altered. The cutting feed rate was reduced to 30 mm/min; the air pressure was decreased to 45 psi; the cutting depth was increased from 0.1 to 0.2 mm; the cutting width was 0.3 mm; and the cutting process involved only down-milling. The reason why the cutting depth and width were increased was that incomplete cutting was observed in cycles 250–300 during the up-milling process. After confirming the cutting conditions and paths, for each cycle starting from the 300th cycle, the removed volume increased to 35.74 ${\mathrm{mm}}^{3}$. Moreover, the time required for the completion of each of these cycles was approximately 47 min. As the cutting experiment progressed, in some cycles, the dental handpiece exhibited the phenomenon of a sudden drop in rotation speed. Although the handpiece appeared to be stable, after approximately 375 cycles, the dental handpiece could not conduct more than 20 cycles of the cutting experiment after a new tooth drill was attached to it. A new tooth drill can typically be used for more than 20 cycles. Therefore, it could be inferred that the dental handpiece had degraded. The cutting experiment of dental handpiece No. 1 was stopped at this moment. This handpiece conducted 416 (250 + 166) cycles of the cutting experiment. The conditions of the cutting experiment for dental handpiece No. 2 were as follows: cutting feed rate = 100 mm/min, air pressure = 50 psi, and cutting depth = 0.1 mm. The cutting process involved linear back-and-forth milling (Figure 7). The cutting width was 0.2 mm until the glass ceramic block was cut to a depth of 0.1 mm. After 250 cycles of the cutting experiment, vibration signals with characteristic frequencies that were not observed under normal conditions gradually appeared; thus, the cutting experiment was terminated.

### 3.2. Free-Running Vibration Signals

**Figure 9:**
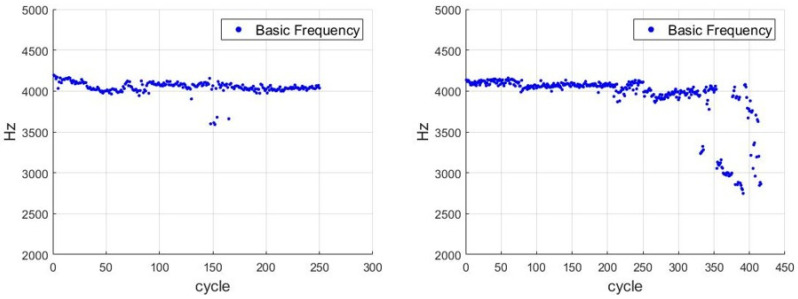
Main rotation speed frequencies of dental handpiece No. 2 (**left**) and dental handpiece No. 1 (**right**).

In [5], a noncontact laser Doppler vibrometer, a condenser microphone, and a mobile Micro Electro Mechanical System (MEMS) microphone were used to measure the vibration signals of a dental handpiece under free running. Under the no-loading (free running) condition, no significant differences were observed in the measurements of the aforementioned instruments. Under in vivo medical conditions, the mobile MEMS microphone was the most suitable instrument for the diagnosis of the health of the handpiece [5]. The authors of [2] used an accelerometer and a laser Doppler vibrometer to measure the vibration signals of a dental handpiece during its operation. The measurement results obtained with these two instruments were plotted into the frequency spectrum. Under the no-loading (free running) condition, the measurement results of these two instruments did not exhibit substantial differences. Therefore, the current study used the FFT to transform the free-running vibration signals obtained from the accelerometer into frequency-domain signals (Figure 9). Dental handpiece No. 1 and dental handpiece No. 2 operated under the same conditions up to 250 cycles. After the 330th cycle, dental handpiece No. 1 began to exhibit problems such as an unstable rotation speed and a sudden decrease in the rotation speed. Moreover, the material removal was incomplete.

**Figure 10:**
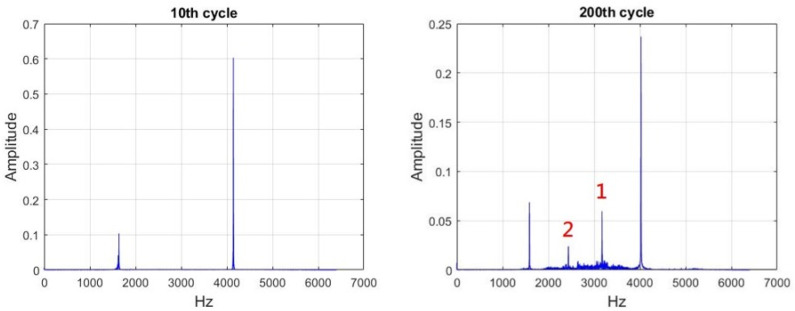
FFTs of the 10th cycle (**left**) and 200th cycle (**right**) for dental handpiece No. 2.

Except for the slight decreases in approximately the 150th cycle, basic frequency (BF) and ball pass frequency of the outer race of dental handpiece No. 2 exhibited no substantial differences throughout the 250 cycles. Figure 9 illustrates the FFTs of the 10th cycle (left) and 200th cycle (right). Figure 10 indicates that during the cutting experiment, the characteristic frequency (CF) of the vibration exhibited two additional subcharacteristic frequencies in each cycle (frequencies labeled 1 and 2 in the right part of Figure 10), and the vibration amplitude gradually increased with the number of cycles. The main frequency (in the left of Figure 10, 4 kHz) gradually decreased as the number of cycles increased. The handpiece components were gradually damaged under a fixed drive energy (50 psi); therefore, other vibration signals were transmitted.

**Figure 11:**
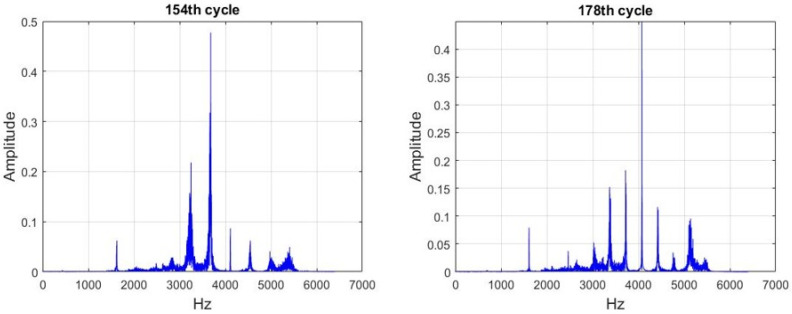
Cycles with unstable frequency domains (the 154th and 178th cycles).

For the frequencies labeled 1 and 2 in Figure 10, CF(1) ≅ 3200 Hz and CF(2) ≅ 2500 Hz. These frequencies were repetitive and noticeable. Therefore, the cycles with an unstable frequency domain in Figure 11 were considered as outliers and removed to facilitate subsequent analysis. A total of 17 cycles were removed. The remaining 233 cycles were used as experimental data to establish the HI and prediction models.

**Figure 12:**
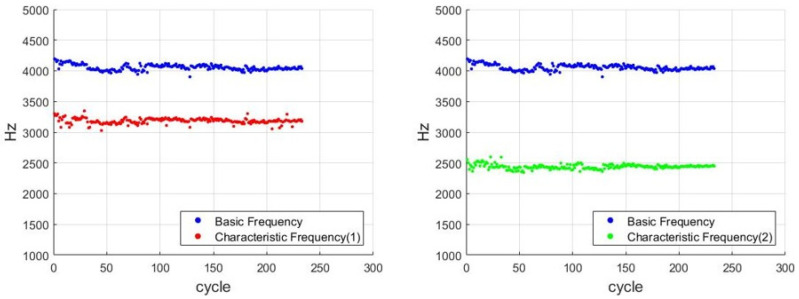
Distributions of the BF, CF1, and CF2 for each cycle.

**Figure 13:**
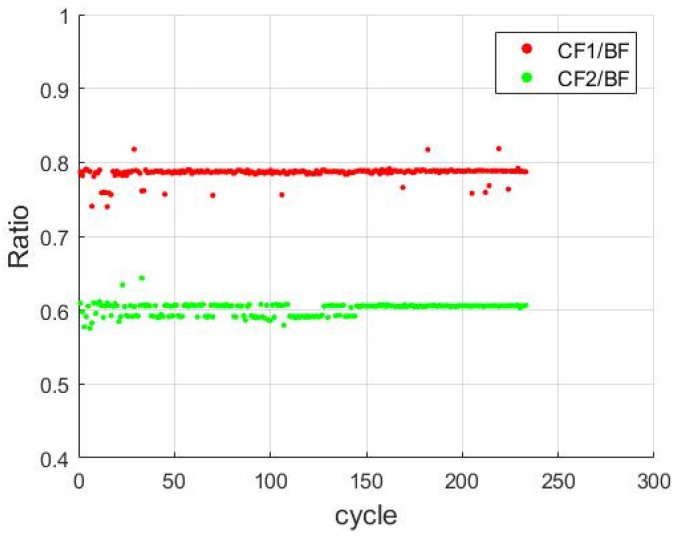
CF1/BF and CF2/BF ratios for each cycle.

Figure 12 illustrates the distribution of CF1, CF2, and the BF for each cycle. As shown in Figure 13 The results indicate that CF1 and CF2 were approximately 78% ($r_{1}$) and 60% ($r_{2}$) of the main frequency, respectively.

To determine whether CF1 and CF2 changed with the progress of the cutting experiment, the trends of these CFs were analyzed by calculating the amplitude ratios of these CFs to the entire frequency domain. The relevant formula is expressed as follows:

(19) $$R\left(r_{j}\right)=\frac{1}{S}\overset{150}{\underset{i=-150}{\sum }}C\left(BF\times r_{j}+i\right),\;j=1,2$$

(20) $$S=\overset{6400}{\underset{k=1}{\sum }}C\left(k\right)$$

where C(x) is the amplitude of the x-section of the frequency distribution of 1–6400 Hz; x is the xth section; r denotes the ration; $r_{1}=0.78$ and $r_{2}=0.6$ for CF1 and CF2, respectively; S is the sum of the amplitude of the entire frequency domain; and R is the ratio of the sum of the CF amplitude to the amplitude sum of the entire frequency domain. The sampling frequency of the capture card adopted in this study was 12.8 kHz. After performing FFT, the study could analyze a frequency of up to 6.4 kHz. Therefore, this study calculated the sum of amplitudes of frequency up to 6.4 kHz. Because the air pressure tube providing air pressure to the dental air turbine handpieces was an elastic plastic tube, the provided air pressure was not completely consistent even under PID control, which resulted in some fluctuation in the CF. Therefore, amplitudes in the frequency ranges of CF1 ± 150 Hz and CF2 ± 150 Hz were summed and used as the CF amplitudes.

**Figure 14:**
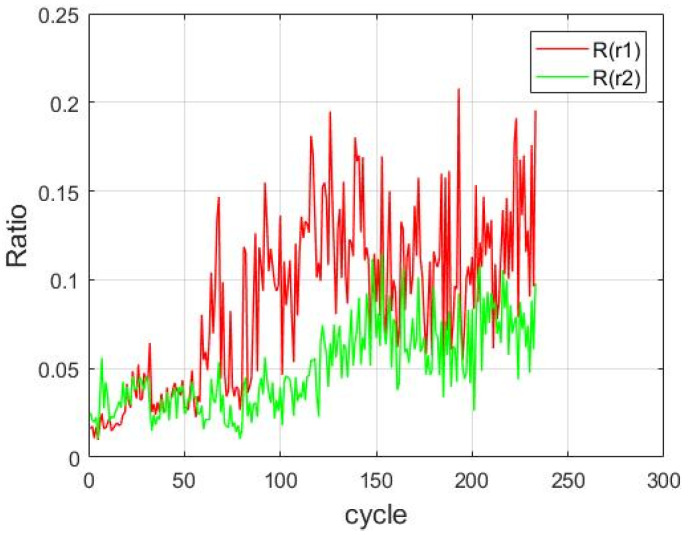
*R* values of CF1 and CF2 in relation to the amplitude of the entire frequency domain.

Figure 14 depicts the *R* values of CF1 and CF2 obtained for each cycle after using (19) and (20). The results indicate that as the experiment proceeded, $R_{\left(r1\right)}$ and $R_{\left(r2\right)}$ substantially increased. These parameters represented CF amplitudes relative to overall amplitudes. Under long-term milling, the amplitude of the CFs of the handpiece components increased; thus, as the number of cycles increased, the dental handpieces were gradually damaged, which resulted in the formation of CFs. Consequently, $R_{\left(r1\right)}$ and $R_{\left(r2\right)}$ are suitable for being used to establish the HI of the dental handpieces.

### 3.3. Building Health Index

**Figure 15:**
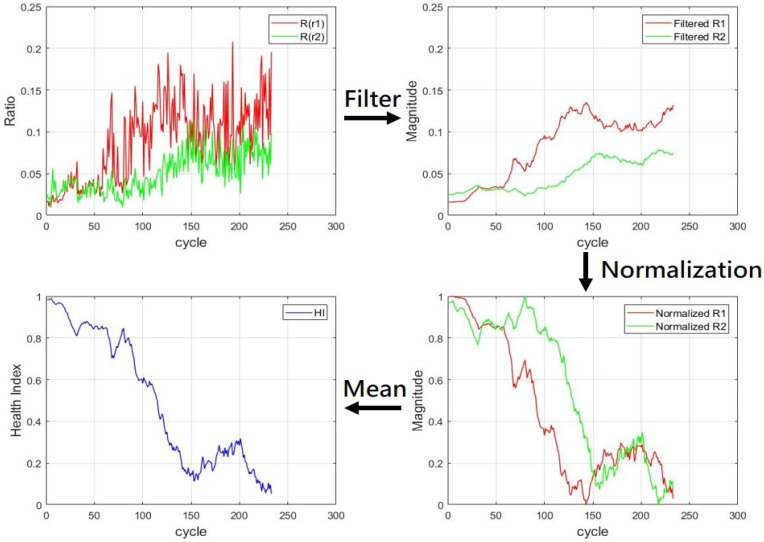
Procedure for generating the HI.

The Kalman filter was applied to $R_{\left(r1\right)}$ and $R_{\left(r2\right)}$ to smooth out the curve in Figure 14 and obtain trend lines in Figure 15 with the coefficients of Q and R by 0.00001 and 0.0013, respectively. The trend lines were normalized to between 0 and 1. All the values were subtracted from the maximum value in the data, which resulted in a reversed trend line, which is represented by (21). In (21), $x_{i}$ represents the data that required normalization, $x_{max}$ is the maximum value in the data, $x_{min}$ is the minimum value in the data, and $X_{n}$ is the normalized data. The aforementioned normalization was conducted to generate an HI in which 1 represented the initial healthy state and 0 represented the most damaged state. The SOH of each cycle was between 0 and 1. Finally, because the two HIs generated from the *R* values were critical indices for the health of the dental handpieces, these HIs were averaged in each cycle to obtain the final HI of the handpieces. Figure 15 illustrates the complete process for generating the HI.

(21) $$X_{n}=\frac{x_{max}-x_{i}}{x_{max}-x_{min}}$$

## 4. Remaining Useful Life Prediction and Degradation Assessment

### 4.1. RUL Based on LSTM Many-to-One Structure

To train and test the LSTM model, odd and even cycles were used as the training and testing datasets, respectively. Therefore, the training and testing datasets comprised 117 and 116 cycles, respectively. The data remained matched to the same cycles after the data segmentation. The model parameters were as follows: the number of time steps was 10, the number of epochs was 100, the loss function was the mean square error, and the number of LSTM hidden layers was 2.

**Figure 16:**
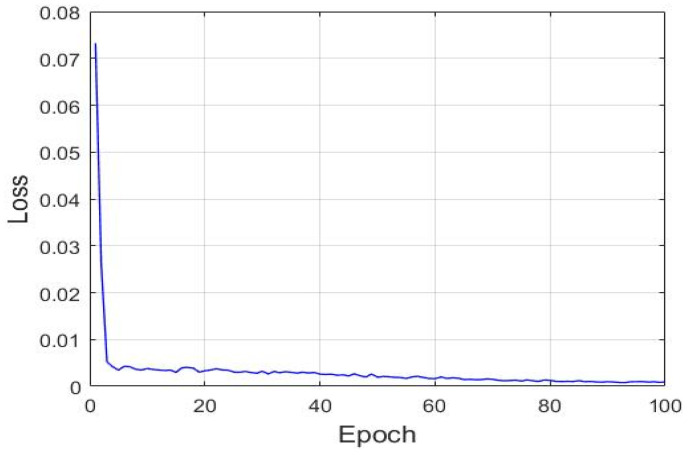
Gradual convergence of the loss function of the LSTM model with the many-to-one structure.

**Figure 17:**
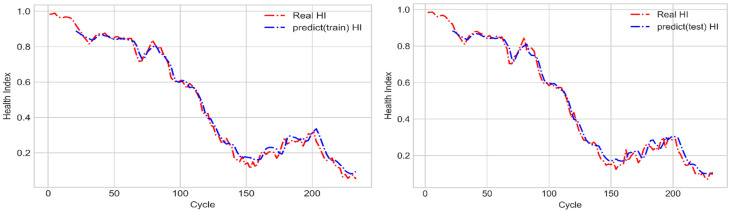
Prediction results obtained with the LSTM model with the many-to-one structure for the training dataset (**left**) and testing dataset (**right**).

An LSTM network with a many-to-one structure was used to establish an assessment model for the condition in which the state of a cycle is known to predict the SOH of the next unknown cycle. This study used the two normalized values as the model input. The two normalized values were from the definition of Equations (19) and (20) for R(r1) and R(r2), respectively. The filtered, normalized and array by two of $R_{\left(r1\right)}$ and $R_{\left(r2\right)}$ data are treated as $x_{\left(t\right)}$ for the LSTM’s manipulation. Therefore, the input data were [$x_{\left(t-18\right)},x_{\left(t-16\right)},x_{\left(t-14\right)}$, …, $x_{\left(t\right)}$], the model output was [$y_{\left(t+2\right)}^{’}$], and the corresponded target HI was [$y_{\left(t+2\right)}$]. After the LSTM model was subjected to training with the training dataset, the mean square error gradually reduced and converged (Figure 16). Figure 17 illustrates the prediction results of the aforementioned model. This figure indicates that after training, the aforementioned LSTM model accurately predicted the SOH even with a dataset unknown to it.

### 4.2. LR Prediction Model

**Figure 18:**
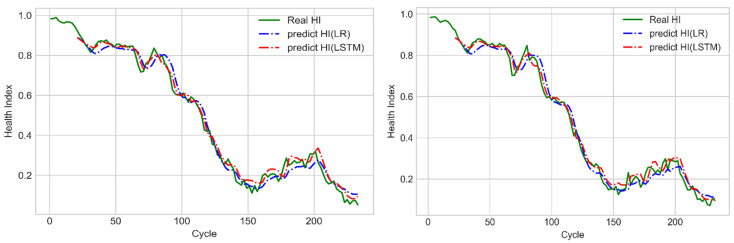
Prediction results of the LSTM model with the many-to-one structure and the LR model for the training dataset (**left**) and testing dataset (**right**).

The LR prediction model was used to establish an assessment model for using known cycle state to predict the SOH of the next unknown cycle. The input data of this model were [$y_{\left(t-18\right)},y_{\left(t-16\right)},y_{\left(t-14\right)}$, …, $y_{\left(t\right)}$], the model output was [$y_{\left(t+2\right)}^{’}$], and the corresponding target HI was [$y_{\left(t+2\right)}$]. After conducting training with the training dataset, the prediction results of the LR model were compared with those of the LSTM model with the many-to-one structure (Figure 18). According to Figure 18, both these models had favorable accuracies. Therefore, the mean square errors of their prediction results were analyzed. As shown in Table 1, the mean square errors of the prediction results of the LSTM model with the many-to-one structure had lower errors than those of the LR model for both the training and testing data.

As previously mentioned, a conventional run-to-failure LR sets y as a constant to 1 for the initial healthy status, to 0 for failure, and to 0–1 for the rest of the work asset’s lifetime. Here, two feature extractions (CF1 and CF2) were fed into the LR model, and resulted in good accuracy. However, the real SOH and RUL of a system were generally unknown in advance, and were changed with time. Raw data for time-forcasting in LR may not apply. In Section 4.1, the LSTM is able to work on the raw sensory data of machine’s condition. The advantage of LSTM is to discover some unseen and hidden structure to improve the general model. The LSTM many-to-one for RUL prediction can do the monitoring job for RUL’s prediction based on few milling process cycles. In Section 4.3, the LSTM’s many-to-many structure will be introduced for the SOH of the next unknown cycles. It is used for the degradation assessment in practice. And it can be deployed whenever one wants to set a threshold for a diagnostic issue or performs a prognostic diagnosis. 

### 4.3. LSTM Many-to-Many Structure

**Figure 19:**
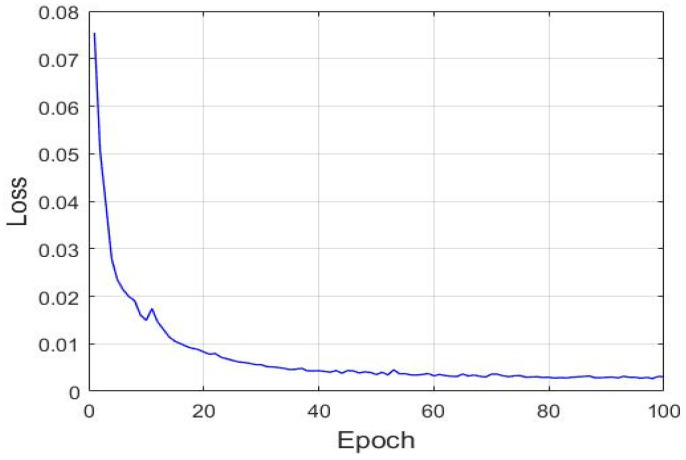
Gradual convergence of the loss function of the LSTM model with the many-to-many structure.

An LSTM model with the many-to-many structure was established for predicting the degradation trajectory of a dental handpiece. In the training stage, the SOH of the known cycle was used as the model input data. Therefore, the input data were [$y_{\left(t-18\right)},y_{\left(t-16\right)},y_{\left(t-14\right)}$, …, $y_{\left(t\right)}$], the model output was [$y_{\left(t+2\right)}^{’},y_{\left(t+4\right)}^{’},y_{\left(t+6\right)}^{’}$, …, $y_{\left(t+20\right)}^{’}$], and the corresponding target HI was [$y_{\left(t+2\right)},\;y_{\left(t+4\right)},\;y_{\left(t+6\right)}$, …, $y_{\left(t+20\right)}$]. During the testing stage, the predicted output of the aforementioned model was used as the input for the next model prediction; thus, iterations were executed for generating the degradation trajectory. After training with the training dataset, the aforementioned LSTM model’s mean square error gradually reduced and converged (Figure 19).

**Figure 20:**
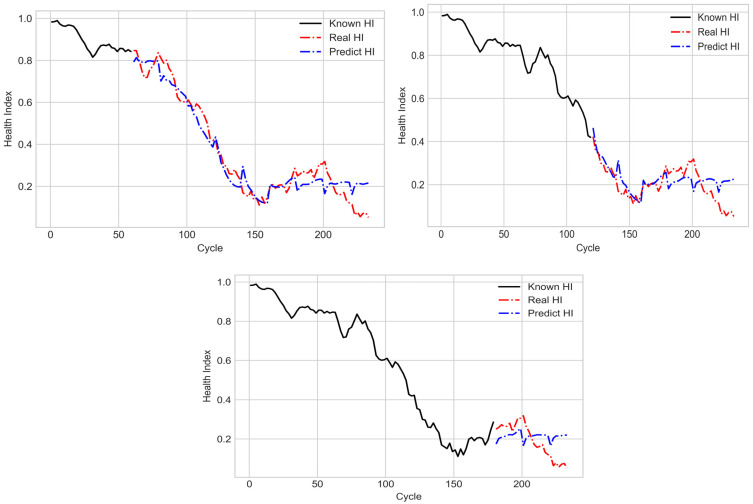
Predicted degradation trajectory for the training dataset under different numbers of known cycles.

**Figure 21:**
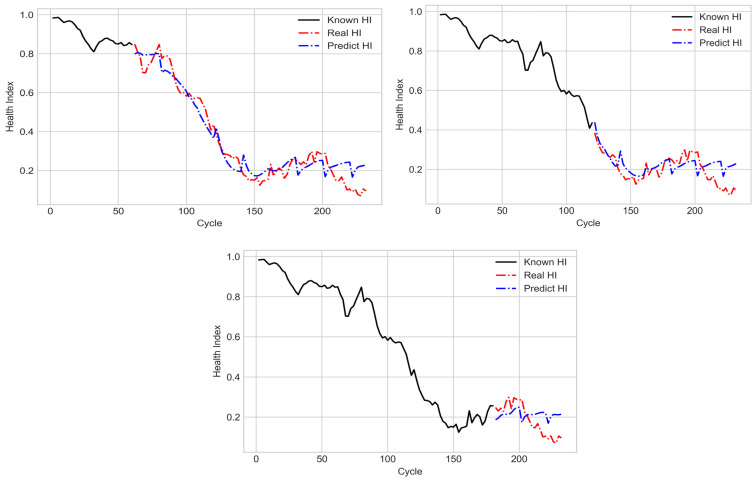
Predicted degradation trajectory for the testing dataset under different numbers of known cycles.

In the testing stage, 30, 60, and 90 known cycles of SOHs were input into the LSTM model with the many-to-many structure for predicting the subsequent degradation trajectory. The results obtained with the aforementioned model for the training dataset are displayed in Figure 20. A note is made here that the trajectories of known HI and real HI in Figure 20 were all from the same conducted experimental results. According to the two figures at the top of Figure 20, when the aforementioned LSTM model was used to predict the degradation trajectory, it reflected a gradually declining SOH in the middle cycles; thus, the aforementioned model achieved favorable prediction results with respect to the target HI. However, in the later cycles, the predicted SOH did not decline; thus, the degradation trajectory did not reflect the target HI well. Figure 21 shows the assessment of the testing dataset by the LSTM model with the many-to-many structure. Although this model was not trained with the testing dataset, the predicted degradation trajectory was similar to that presented by the training dataset. Table 2 indicates that when the number of known cycles was 90, the prediction results for both the training and testing datasets exhibited the largest mean square errors. This result was obtained because the aforementioned model could not favorably predict the SOH in later cycles, which resulted in a large error. Additionally, the two datasets were subsets of the same dataset; therefore, the mean square error of the testing dataset was lower than that of the training dataset.

## 5. Conclusions

The conclusions of this study are as follows:

1. This study used a triaxial accelerometer to measure the vibration signals of dental handpieces under the free-running condition. The collected data were subjected to the FFT, after which the frequency spectrum of the rotation speed was plotted. Subsequently, the amplitudes of CFs and the main rotation speed frequency were identified in the frequency domain and compared to obtain two critical CFs related to the SOHs of the dental handpieces. These CFs were used as indices for predicting the RUL and assessing the degradation of the handpiece.
2. This study established an LSTM model with the many-to-one structure that can store and transfer memories and an LR prediction model that cannot store memories to predict the SOHs of the dental handpieces. These models exhibited favorable prediction results. However, the LSTM model had a lower mean square error than the LR model did, which indicates that the LSTM model could monitor the SOH for a long time. The aforementioned LSTM model can be used to predict the RULs of clinical dental handpieces with high accuracy.
3. An LSTM model with the many-to-many structure was used to predict the SOHs of unknown cycles. The obtained output was then used as the input for the next unknown cycle; thus, iterations were executed. Although the aforementioned LSTM model did not provide highly accurate results for the later cycles (with respect to the real HI), the model could favorably reveal the gradual degradation of the dental handpieces in the middle cycles.

## Figures and Tables

**Table 1:** Mean square errors of the prediction results of the LR model and the LSTM models with the many-to-one structure.

	LSTM	LR
Training dataset	0.000769	0.001359
Testing dataset	0.000820	0.001360

**Table 2:** Mean square errors of the prediction results of the LSTM model with the many-to-many structure under different numbers of known cycles.

	30 Known Cycles	60 Known Cycles	90 Known Cycles
Training dataset	0.006889	0.005170	0.009435
Testing dataset	0.005132	0.004260	0.007701
